# The STRAND Chart: A survival time control chart

**DOI:** 10.1002/sim.8065

**Published:** 2018-12-26

**Authors:** Olivia AJ Grigg

**Affiliations:** ^1^ MRC Biostatistics Unit University of Cambridge Cambridge UK

**Keywords:** control chart, performance monitoring, quality control, risk adjustment, survival

## Abstract

The STRAND Chart (Survival Time, Risk‐Adjusted, N‐Division Chart) is a new tool for online risk‐adjusted (RA) monitoring of survival outcomes. The chart is drawn in continuous time, making it responsive to change in the process of interest, for example, performance over time of a surgical unit and the procedures that they employ. Though it is difficult to achieve with charts designed for the purpose described, we show that our suggested chart keeps patient ordering intact. We discuss the difficulties maintaining patient ordering poses, making reference to other charts in the literature. Our conclusion is that the best approach to preserving patient ordering on any chart of this nature involves compromising on the fullness of presentation of the recorded data. The chart is divided into N strands, each strand relating to a benchmark patient's survival information at t_n_ days following treatment, n = 1,2,…,N. We present a simple version of the chart where the strands consist of Bernoulli RA exponentially weighted moving averages, recording RA failure rates at t_n_ days. It can detect recent process change and historical change. We illustrate the STRAND Chart using a well‐known UK post cardiac surgery survival dataset, where the nature of a certain cluster in the data can be seen.

## 1 INTRODUCTION

Monitoring of patient outcomes over time is prolific in the field of medicine, most especially in the form of sequential clinical trials.^1^, ^2^, ^3^ Outside of the clinical trials setting, there has been increasing interest in assessing quality of care provided, where there are potential underlying changes in performance and also planned changes in care that are not formally assessed by clinical trial (such as periodic retraining of surgeons).^4^, ^5^, ^6^, ^7^ While quality control and the use of control charts for process monitoring has its roots in the industrial setting where monitored items, for example, electrical components, are homogeneous in nature,^8^, ^9^ in the medical context, the “items” monitored (patients) are heterogeneous in nature.^10^, ^11^, ^12^ Patients have different underlying characteristics and morbidities that, though they affect outcomes, are beyond the control of the medical practitioner or unit and hence need to be adjusted for. This aspect of monitoring in medical contexts has given rise to a growing body of work on risk‐adjusted (RA) monitoring with control charts.^10^, ^11^, ^12^, ^13^, ^14^, ^15^, ^16^, ^17^, ^18^, ^19^, ^20^

In the particular area of RA monitoring of survival data (akin to reliability or lifetime data in the industrial setting),^21^, ^22^ development of methodology has moved from monitoring in discrete time to monitoring in near‐continuous time, at finer timescales. Where more basic methods record outcomes after some fixed period of time (30 days say) has elapsed, methods developed more recently react to survival events as and when they occur.^10^, ^14^, ^15^, ^16^ Though they are not strictly in continuous time, we will henceforth refer to near‐continuous methods as continuous.

All of these methods fall broadly into two types: those that use sequential log‐likelihood ratio (LLR) statistics and those that use smoothed estimators of some summary statistic of survival such as survival beyond 30 days, mean or median survival. The first type are cumulative sum (CUSUM) charts^23^ that are cumulatively summed LLR statistics of a null process state versus an “out‐of‐control” process state, where an out‐of‐control process may have an elevated median survival for example. The second type are charts that smoothly estimate some summary statistic of survival, an example being exponentially weighted moving average (EWMA) charts.^24^, ^25^ These EWMA charts can be constructed from a Classical standpoint or from a Bayesian standpoint, where the former treats the chart measure as a statistic with sampling error and the latter treats it as a posterior estimate for a randomly wandering underlying process parameter.^11^, ^17^ Where Classical EWMA charts and CUSUM charts are set up using operating characteristics such as in‐ and out‐of‐control average run lengths to a signal of process change,^26^, ^27^ Bayesian EWMA charts would focus on quality of estimation and hence on width and placement of credible intervals.^11^, ^17^, ^28^, ^29^, ^30^, ^31^, ^32^, ^33^, ^34^ The width of the credible intervals about the chart statistic determines how often the chart will signal and the placement determines the balance between signals of improvement and signals of deterioration in the process. As with Classical charts, Bayesian EWMA charts and similar Bayesian time series charts would be tuned on test data before being applied to online data.^35^ Tuning the chart to the pilot data can be viewed as an exercise in analysis of sensitivity to changes in chart parameters.

The charts are based on varying degrees of parameterisation of the survival curve. While all methods use a fully parametric risk model, some methods specify the survival curve fully and others specify it only in part. We refer to the latter as semiparametric charts in this context.

In Section 2, we review existing charts in the literature designed to monitor RA survival outcomes. In Section 3, we outline the details of our chart, and in Section 3.1, we apply the chart to the now classic post cardiac surgery survival data from a UK hospital, 1992‐1997.^11^, ^12^, ^14^, ^15^, ^17^, ^18^ In Section 4, we compare the performance of our chart to that of the best competing chart in the literature.

## 2 REVIEW OF RISK‐ADJUSTED SURVIVAL TIME CONTROL CHARTS

### 2.1 The RA CUSUM and RAST CUSUM

These methods capture different aspects of the survival process being monitored. The RA Bernoulli CUSUM of Steiner et al accumulates information about the measure Pr(*X* ≤ *d* number of days) where *X* is a dynamic censored survival process.^12^ The information is gathered via a cumulatively summed (CUSUM) LLR statistic of a null risk model versus an alternative model where the failure rate before *d* number of days is assumed to be higher (or, alternatively, lower) across all patients. An LLR CUSUM can be written as

(1) $$C_{t+1}=C_{t}+LLR_{t},\;t=1,2,\text{…},$$

where *t* indexes arriving data.

The risk‐adjusted survival time (RAST) CUSUM of Sego et al similarly accumulates an LLR CUSUM statistic of a null versus an alternative hypothesis,^15^ but the likelihood used reflects the whole parametric survival model *f*_*X*_ (*x*) as opposed to a prespecified tail of the survival distribution Pr(*X* ≤ *d*). Hence, the RAST CUSUM of Sego et al is fully parametric, whereas the RA Bernoulli CUSUM of Steiner et al is semiparametric.

The charts, with their differing specification of the likelihood, are presented as discrete time methods. They are updated after a fixed period of time, *d* days, has elapsed following treatment. They have been presented in application to the now classic UK cardiac surgery dataset of 1992‐1997, where *d*= 30‐day survival post cardiac surgery is of interest. An application of the STRAND Chart (Survival Time, Risk‐Adjusted, N‐Division Chart) to this same dataset is given in Section 3.1.

These charts, as discrete‐time charts, are not as responsive to the continuous process as they might be, being slow to react to early failures. A move from discrete‐time charts to continuous‐time charts, then, would lead to an improvement in efficiency in detecting process change.

Methods developed subsequently have moved to continuous‐time monitoring of survival data, where failures are recorded as and when they occur rather than being recorded *d* number of days after treatment. Since they update on a finer timescale, these charts are more reactive to deterioration in the survival process.

A challenge for continuous‐time survival time control charts is the problem of maintaining patient ordering, so that time ordering according to the treatment timeline is preserved. The difficulty lies in the fact that patients do not fail in necessarily the same order that they arrive in. If patient ordering is maintained by a chart, clusters of unusual failures or successes in cohorts of patients on the treatment timeline can be identified by the chart effectively. Another challenge is presenting the data in a sensible and interpretable way. We believe that there is no perfect solution to creating a (risk‐adjusted) survival time control chart that meets both of these criteria and that the best solutions given the first challenge is met will need to compromise on the fullness of presentation of data.

The reason survival time charts began as discrete as opposed to continuous is that, though recording in continuous time would on the surface appear quite feasible, updating in discrete time avoids corruption of patient ordering on the timeline. If failures were charted when they occurred instead of at the end of the censoring window, the ordering on the chart would then be by outcome day rather than surgery day. As a result, a significant and important localised change on the surgery timeline might be masked on the chart.

### 2.2 RA CUSUM in continuous time based on the Cox model

This RA CUSUM in continuous time of Biswas and Kalbflesich records patient survival time data in continuous time as and when updates come in, by way of a Cox proportional hazards survival model.^16^, ^36^

The chart is an LLR CUSUM, testing for a fixed change across patients in the instantaneous hazard, or intensity (the probability of imminent failure). The chart is semiparametric in nature since, though the risk model is fully parametric, the survival curve itself is left unspecified.

The continuous‐time passage of patients through the censoring window is regularly recorded on the chart. Outside of these regular records, the chart is updated whenever a failure occurs. Since information is recorded as and when it arrives and is not reshuffled according to patient order, any clusters of failures or long ongoing survivals on the treatment timeline may be contaminated by noise and potentially not detected by the chart. A remedial step would be to adopt the Weighted CUSUM approach of Yashchin,^21^, ^37^ weighting data as it comes in, giving heavier weights to more recent patients. This would lead to a CUSUM method akin to the updating EWMA (uEWMA) Chart of Steiner and Jones discussed in the next section,^14^ or the chart could be drawn reshuffled according to patient order, leading to a method akin to the Improved Bernoulli CUSUM of Keefe et al discussed in Section 2.4.^10^ Such a chart would need to be regularly redrawn on new axes however. We elaborate on this in Section 2.4.

### 2.3 uEWMA for survival time data and Weighted CUSUM

This method of Steiner and Jones, as with a Weighted Biswas and Kalbfleisch CUSUM, does respect patient ordering by weighting incoming survival data (failures or ongoing incrementing survival) according to how recently a patient was treated.^14^, ^16^ It exponentially weights the *j*th patient's current contribution *S*_*j*,*t*_ to the user's chosen survival summary statistic (the choice allowing for both fully parametric and semiparametric methods) according to where that patient is on the current list of patients treated *j* = [1,…,*J*_*t*_]. Patients operated on longer ago are downweighted by a factor (1 − *k*)^*r*^ according to the distance on the list between them and the most recent patient. The uEWMA chart can be written as

(2) $$S_{t}=kS_{J_{t},t}+k(1-k)S_{J_{t}-1,t}+k{\left(1-k\right)}^{2}S_{J_{t}-2,t}+\text{…}+k{\left(1-k\right)}^{J_{t}-1}S_{1,t}\;t=1,2,\text{…},\;0<k<1.$$

The chart effectively damps older cohorts of data down in the current cross‐section of data at time *t*, lessening the effect of the disorder caused by patient failures not occurring in the same order as arrivals. In practice, especially for larger *k*, the weights decay quickly, so that the chart statistic *S*_*t*_ is little affected by only taking the summation over the most recent block of patients.

In damping the older data down, the chart's sensitivity to historical change is reduced. Its focus is drawn toward short‐term behaviour of the process, rendering it more reactive to deterioration in surgical performance and less reactive, in particular, to improvements further back relating to long survivals of higher‐risk patients. This is a drawback of a method such as this that respects patient ordering via the use of decaying weights. Valuable information in the data regarding older cohorts is downgraded.

Another method that weighs incoming data in this way is the Weighted CUSUM of Yashchin.^21^, ^37^ Yashchin remarks that their approach as applied to survival time (equivalently lifetime, or reliability) data is not “sufficient [alone] for efficient detection of change in the process level” and that “supplementary tests may be needed.”^22^ We suggest that rather than introducing such supplemental tests to make up for the limitations of the chart employed, the chart design should be reconsidered. We present our proposed method in Section 3. In the next section, we review a method that maintains patient ordering while accounting for ongoing survival in a conservative way.

### 2.4 Improved Bernoulli CUSUM

This chart of Keefe et al is an improvement to the Bernoulli RA CUSUM of Steiner et al in that failures are recorded as and when they occur as opposed to when a fixed censoring time has elapsed.^10^, ^12^ The chart is redrawn at the occurrence of new failures in such a way that patient ordering is respected. Ongoing patient survival is conservatively treated in that patients yet to have, if at all, recorded failures are assumed up until that time to be cases that survive past the censoring time *d*.

This chart picks up process deterioration faster than the standard Bernoulli CUSUM because failures are recorded when they occur. However, a chart that did not employ such conservative treatment of patients yet to present as failures would identify process deterioration faster still. If full survival information were recorded for patients continuing to survive, rather than the conservative measure used, the chart would be more efficient, both for detecting process deterioration and improvement. This use of full survival information would make it akin to the Biswas and Kalbfleisch CUSUM,^16^ but with reshuffling of points on the chart at regular redrawing of the chart, so that the chart adheres to patient ordering.

As we believe is necessary for a (risk‐adjusted) survival time control chart, the chart does compromise on fullness of presentation of the data in that it requires being redrawn on a new set of axes as new failures occur. We consider the chart to be well suited to examining how clusters develop in a snapshot of time. However, we think it is not suited to viewing clusters and clusters of clusters in bigger frames of time, unless an additional low signal boundary were set and clusters of signals above that low boundary were recorded on a separate chart. The redrawing of the chart is not so much of a drawback when the censoring window is fairly short, as for the post cardiac surgery data (where *d* = 30 days), since the chart is fixed once a lag the size of the censoring period has elapsed. However, it is more of a drawback when the censoring window is of the order of years, as for example, with the transplant data analysed by Biswas and Kalbfleisch^16^: one has to wait a year, say, before the plotted chart at least a year previous to that is no longer subject to change.

## 3 METHOD INTRODUCED HERE: THE STRAND CHART

We propose a new method, the STRAND Chart, which is our solution to survival time monitoring where patient ordering is maintained and where balance between early failures and longer‐term survivals within data cohorts is maintained. The chart can be a Bayesian or Classical chart. We present it as a Bayesian chart as we see this as the more natural approach. The theoretical origins of the chart are fairly complex but the resulting method is simple to implement and interpret.

If we consider a survival time distribution where patients survive a number of days following an intervention such as surgery, the STRAND Chart can estimate probabilities of failure at each of *t*_1_,*t*_2_,…,*t*_*N*_ days. The first strand estimates the failure rate at, say, *t*_1_ = 1/2 a day, the second the failure rate at, say, *t*_2_ = 2 days and so on. The last strand would, we suggest, relate to the failure rate at the censoring time of, say, *t*_*N*_ = 30 days. We refer to survival past *t*_*n*_ days (*n* in 1,…,*N*) as survival past “gate” *g*_*n*_. In the example application in Section 3.1, these failure rates are small, as one would expect from a high quality process. The chart signals when the credible interval about any strand excludes a target value, for example, the estimated failure rate from the pilot data.

**Figure 1:**
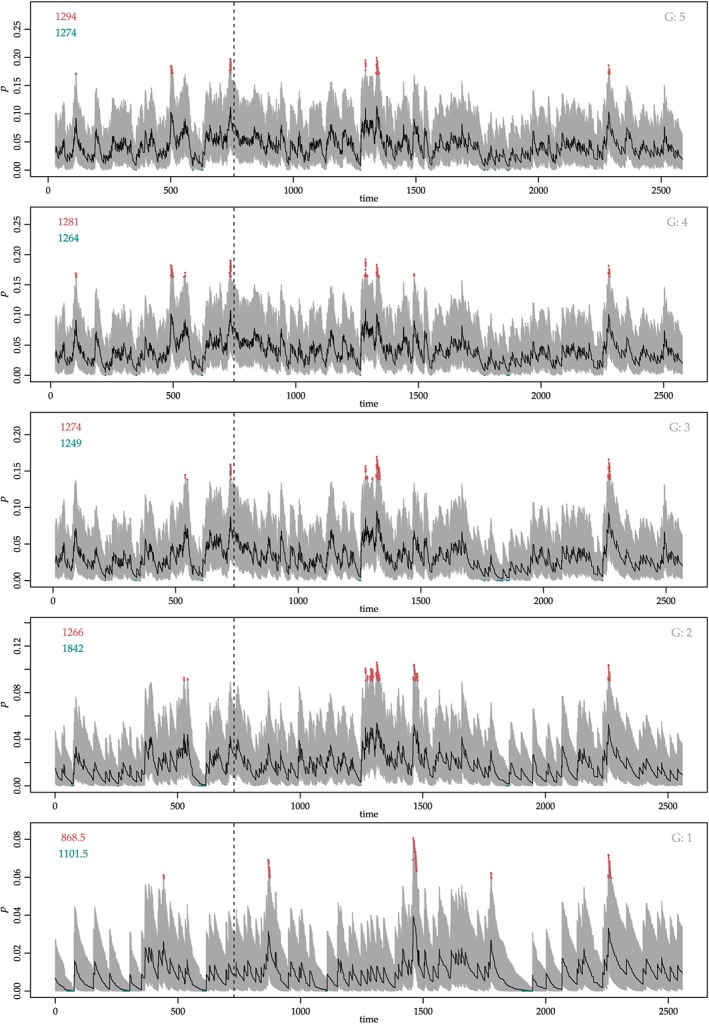
STRAND Chart (Survival Time, Risk‐Adjusted, N‐Division Chart) showing dynamic risk‐adjusted survival rate estimates for gates at (1/2, 2, 10, 20, 30) days, 1 Jan 1992 to 31 Dec 1997. First upper and lower signals respectively on each strand shown in upper left corners. Key:  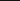  rate;  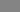  95% credible interval;  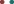  signals of rise/fall in rate;   ‐ ‐ ‐   pilot data cut‐off [Colour figure can be viewed at wileyonlinelibrary.com]

The discretisation into *N* strands means that the chart is not fully continuous, though increasingly fine continuity can be achieved by adding more strands to the chart. Too many strands increase the complexity of the chart and so reduce human readability. Also, too many strands can result in a reduction in chart efficiency (see Section 4). However, with more strands, the passage of unusual cohorts of patients can be seen to track diagonally upward through the chart, by way of peaks and troughs, in a ripple effect. The nature of any clusters can be seen to play out (see Figure 1): a cluster of late failures in a cohort of patients would affect only upper strands of the chart; clusters of early failures might be balanced in a cohort by unusual longer‐term survivals, so that an effect on lower strands would be nullified in upper strands. That the chart allows for the equal balance of failures and survivals in cohorts of data is an important feature.

As a Bayesian chart, the STRAND Chart can be formed of Bayesian time series estimators, updating estimates of the probability of failure at each gate *g*_*n*_, *n* = 1,2,…,*N* in the light of the data. We present the chart here as a set of Bayesian Bernoulli risk‐adjusted exponentially weighted moving average (RA EWMA).^17^ The RA EWMA emanates from a model where the probability of failure at time *i*, *μ*_*i*_, satisfies the following conditions:

(3) $$\begin{array}{ll} E[\mu_{i}] & =E[\mu_{i-1}]\\ P[\mu_{i}] & =\kappa P[\mu_{i-1}], \end{array}$$

where *E* represents a priori expectation and *P* represents a priori precision, the latter being equal to one over the variance and a priori meaning before the data point *y*_*i*_ is seen. According to the model, the quantity *μ*_*i*_ is said to be “mean steady” and is assumed to have decaying precision *P*, by a factor *κ*, from one time point to the next. It is assumed to change smoothly with time and this smooth process can be learnt about via the data *y*_*i*_, *i* = 1,2,….

From the mean steady model, the EWMA statistic

(4) $$m_{i}=\kappa m_{i-1}+(1-\kappa )y_{i};\;i=1,2,\text{…};\;0<\kappa <1$$

results as the posterior mean for *μ*_*i*_ and the associated precision *P*[*μ*_*i*_] tends to 1/(1 − *κ*) as *i* tends to infinity.^17^

As an additional step, to account for patient heterogeneity, risk adjustment of the EWMA can be made so that we have

(5) $$m_{i}=\kappa m_{i-1}+(1-\kappa )[y_{i,j}-{\hat{\mu }}_{i,j}+{\hat{\mu }}_{i,b}];\;i=1,2,\text{…};\;j=0,1,2,\text{…},b,\text{…},J;\;0<\kappa <1,$$

where the *i*th outcome at a gate, relating to a patient of type *j*, is approximately adjusted to the metric of a baseline patient (one with a benchmark risk value) of type *j* = *b* via a risk model relating the expected rate for the arriving patient *μ*_*i*, *j*_ to the current expected rate for a baseline patient *μ*_*i*,*b*_. This risk adjustment step is elaborated on in the application section, Section 3.1. Through the risk adjustment step, *m*_*i*_ becomes an estimate for *μ*_*i*,*b*_, which we refer to as simply *μ*_*i*_.

As discussed elsewhere,^11^, ^17^ these RA EWMAs can also be applied from within the frequentist framework. In the Bayesian approach, we note that there is formal admission that there is uncertainty about the first point on the chart, *m*_0_.

The uncertainty about the RA EWMA statistic *m*_*i*_ is accounted for as follows. Bounds are placed about *m*_*i*_ to express uncertainty about the baseline mean *μ*_*i*_ = *μ*_*i*,*b*_. Since the data are Bernoulli, we suggest for simplicity that a conjugate Beta distribution be chosen. We also suggest, again for simplicity, that the precision at time *i* be set to the steady‐state value 1/(1 − *κ*) so that the bounds about *m*_*i*_ are percentiles of a Beta distribution with mean *m*_*i*_ and precision 1/(1 − *κ*).

In summary, all that is required to apply the essential form of the STRAND Chart is a risk model relating patients to a baseline or benchmark patient, a choice of gates, a choice for *κ* (which we will elaborate on in the application section, Section 3.1), and outcomes *y*_*i*, *j*_ indicating whether the *i*th patient survives or fails at a gate along with risk scores *z*_*i*, *j*_ for those patients. Only the data are required, a choice of gates and an indication of the amount of data to be apportioned as pilot data, to implement the essential form of the STRAND Chart using the R package “strand” (available from the author).

It should be noted that there are existing Bayesian methods for monitoring survival data.^38^, ^39^, ^40^ However, these focus on retrospective changepoint estimation and, by this, contrast with online monitoring methods. Where online methods are ideal for detecting process change forward in time, changepoint methods are ideal for locating with hindsight when that change occurred.

### 3.1 Example: post cardiac surgery survival

There is an accompanying package in R, “strand,” see Supporting information, for users to implement the chart. We used the package to draw a chart of the now classic UK cardiac surgery data of 1992‐1997, treating, as in other papers, the first two years' data as pilot data.^14^, ^15^ We chose gates at (1/2,2,10,2,30) days with the aim of giving a comprehensive yet not too detailed cross‐section of the overall survival distribution. Patient risk is summarised by the Parsonnet score,^41^ and survival is censored at 30 days following surgery. The resulting chart can be seen in Figure 1.

The risk model, assumed to be a set of logistic models, one for each strand, is calibrated to pilot data. This means that the chart adopts an empirical Bayes approach where the initial prior for the variable of interest is calibrated on pilot data. For each strand, this provides a starting point *m*_0_ for the incrementing estimate, *m*_*i*_, of the baseline failure rate *μ*_*i*_ at a particular gate.

The STRAND Chart could alternatively be initialised by fitting an overarching fully parametric survival model as opposed to a separate model for each strand. However, fitting separate risk models for each strand allows the survival distribution to go unspecified, resulting in a semiparametric method that is more flexible.

The logistic risk model assumes that the odds of failure at a gate for a patient of type *j* is linked to the odds of failure for a baseline patient by a scaling factor, such that

(6) $$\left(\frac{{\hat{\mu }}_{j}}{1-{\hat{\mu }}_{j}}\right)=\left(\frac{{\hat{\mu }}_{b}}{1-{\hat{\mu }}_{b}}\right)e^{\hat{\beta }(z_{j}-z_{b})},$$

where *z*_*j*_ is the patient risk score, in this case the Parsonnet score, and *z*_*b*_ is the baseline patient risk score. As per the paper of Steiner and Jones, we take the baseline Parsonnet score to be the median in the pilot data, *z*_*b*_ = 7. For these data, Equation (6) gives $\hat{\beta }=(0.067,0.071,0.074,0.074,0.077)$ for the strands relating to the survival rates at (1/2,2,10,2,30) days.

When a patient of type *j* arrives at any of the gates at time *i*, their binary outcome of success is noted. The risk adjustment step can be carried out by assuming that the current predicted failure rate at that gate for a baseline patient, ${\hat{\mu }}_{i,b}$, is equal to the previous chart value *m*_*i* − 1_. Substituting this into (6) gives

(7) $${\hat{\mu }}_{i,j}=\frac{\left(\frac{m_{i-1}}{1-m_{i-1}}\right)e^{\hat{\beta }(z_{j}-z_{b})}}{1+\left(\frac{m_{i-1}}{1-m_{i-1}}\right)e^{\hat{\beta }(z_{j}-z_{b})}}.$$

By using the logistic risk model, differing patient risks can be predicted by the STRAND Chart simply by scaling up or down on the odds scale by the factor $e^{\hat{\beta }(z_{j}-z_{b})}$.

Given the vector of binary outcomes at time *i*, *y*_*i*, *j*_, ${\hat{\mu }}_{i,b}=m_{i-1}$ and ${\hat{\mu }}_{i,j}$ as given by (7), $y_{i,j}-{\hat{\mu }}_{i,j}+{\hat{\mu }}_{i,b}$ is the RA data value to be fed into the RA EWMA.

The (vector‐valued) smoothing parameter *κ* can normally be chosen by minimising the mean squared error of prediction in the pilot data. However, since this curve tends to flatten out for *κ*→1 for Bernoulli data, we recommend choosing a *κ* that is large enough for good prediction (low mean squared error) yet small enough to allow the chart to be reactive to change in the process. We recommend using the following formula as a guide:

(8) $$\kappa =0.993-0.192m_{0},$$

where *m*_0_ is the starting value for a particular strand. This was developed from the example data, so is most useful for applications with similarly small failure rates *μ*. For these data, Equation (8) gives *κ* = (0.992,0.990,0.987,0.986,0.985) for the strands relating to survival at (1/2,2,10,20,30) days, respectively.

The Bayesian version of the chart allows the chart statistic to be treated as the posterior mean of the rate of failure for a baseline patient at the survival gates, *μ*_*i*,*b*_. The steady‐state Bayesian RA EWMA allows the precision of the posterior distribution for *μ*_*i*,*b*_ to be fixed at 1/(1 − *k*). Credible intervals around the RA EWMA estimate for *μ*_*i*,*b*_, *m*_*i*_, act as control limits for the chart. These can be set by adjusting the probability interval. The default interval in the strand package is a symmetric 95% credible interval.

In terms of placing the credible intervals, we do not recommend doing this by running simulations under null and alternative hypotheses where the true mean is fixed and considering run length properties. To tune to test criteria, we recommend setting the posterior credible bounds to the default and if necessary widening or narrowing one or both so that no or very few signals occur in the pilot data. Credible interval width and placement seems a more natural and simpler consideration to us than considering run length properties under basic null and alternative hypotheses. In the placement of the credible intervals, the user can make the chart as robust as they require using the pilot data as a guide. We chose the interval width and placement to give signals in the pilot data similar to those seen in other applications to these data in the literature,^12^, ^14^, ^15^, ^17^ that is, few signals of process improvement or deterioration.

Though we do not recommend the consideration of run length properties of the chart for setting chart parameters, it is nonetheless of interest to consider the performance of the STRAND Chart compared to competing charts, appealing to run length characteristics as standard in the literature.^10^, ^14^ In Section 4, we compare the chart to the best‐performing chart in the literature, the uEWMA.^14^

To construct the posterior credible interval for *μ*_*i*,*b*_, we can assume a conjugate Beta(*m*_*i*_/(1 − *κ*),1/(1 − *κ*)) distribution whose mean is *m*_*i*_ and whose precision is 1/(1 − *κ*). For 95% credible intervals, we take an interval of width 95% from this Beta distribution. For this application, we chose asymmetric bounds with the lower boundary at 5% and the upper boundary at 97.5%, increasing signals of deterioration compared to symmetric bounds of 2.5% and 97.5%. Choosing these bounds gives a similar signalling pattern to other charts in the literature applied to this particular dataset.^12^, ^14^, ^15^, ^17^

In terms of results from the data, new information is available about these data when implementing the STRAND Chart. In particular, the cluster of unusual deaths around three and half years (1266 days) from the start of monitoring can be analysed in more detail: the nature of the cluster can be seen to play out in Figure 1. There are no signals at that particular period of time at the 1/2‐day survival gate (the lowest strand). At the 2‐day survival gate (the next strand up), a block of red indicating signals is evident, starting at 1266 days. As longer‐term survivals are weighed in, however, at the 10‐ and 20‐day gates (the next two strands up), the blocks of red reduce in size and the extremity of the overall signal of a cluster is reduced. This equal balancing of failures and survivals in cohorts of data that the STRAND Chart allows is an important feature.

## 4 CHART PERFORMANCE

The best‐performing chart in the literature appears to be the uEWMA with LLR scores.^14^ We compare this chart, through consideration of run length properties under basic null and alternative hypotheses, to a STRAND Chart with the same parameters as that in Figure 1. We also introduce into the comparison the uEWMA with scores relating to 30‐day survival as well as a STRAND Chart with a single gate estimating 1/2‐day survival.

**Figure 2:**
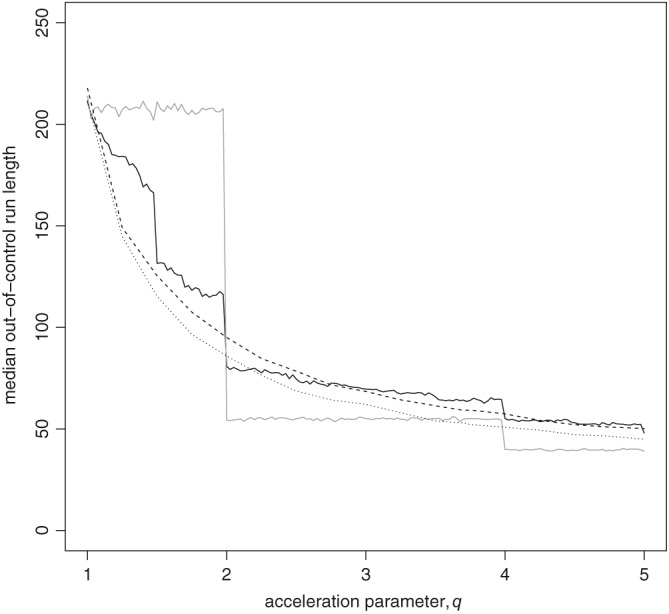
Efficiency of STRAND Chart (Survival Time, Risk‐Adjusted, N‐Division Chart) versus uEWMA (updating exponentially weighted moving average) for the example data as measured by median “out‐of‐control” run length for a given survival acceleration q. Key:  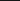  STRAND Chart as per Figure 1;  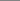  STRAND Chart with 1 strand at 1/2 day;  ⋯  uEWMA with log‐likelihood ratio weights;  ‐ ‐ ‐  uEWMA with 30‐day survival weights

The simulations upon which the performance is assessed were carried out via empirical bootstrapping from the example data, that is, sampling without replacement from the example data pairs of survival times and Parsonnet scores. For the “in‐control” scenario the data were sampled without any alteration. For the “out‐of‐control” scenario, the bootstrapped survival times were accelerated by a factor *q*, as per the paper of Steiner and Jones.^14^ Figure 2 shows the results. All simulated values were based on 20 000 chart runs. As a performance measure, median run lengths were estimated.

It can be seen from Figure 2 that, for survival times shortened by a factor *q*, the uEWMA with LLR scores appears to be more efficient than the STRAND Chart in the format of that in Figure 1 for most values of *q*. The uEWMA with scores relating to 30‐day survival, however, is sometimes more and sometimes less efficient than that STRAND Chart. Contrastingly, the STRAND Chart with a single gate at 1/2 a day is sometimes more and sometimes less efficient than the uEWMA with LLR scores. It seems that a STRAND Chart with a single early gate may be more efficient than one with multiple gates, though, due to the step‐function nature of the median run length function, a STRAND Chart with a single early gate may not be sensitive to small changes in the survival distribution compared to other charts.

Although having a single early STRAND might improve efficiency for moderate to large values of *q*, less can be deciphered about the monitored process by just having one early strand. In reality, process changes are likely to be more complex than a simple acceleration of survival times such that the survival curve is affected in different ways. Hence, we recommend having more strands to capture more information about the process as it changes with time and to capture more information about any clusters of failures. The potential sacrifice of efficiency we believe is worth the gain in information.

Note that, for the STRAND Chart with gates at (1/2,2,10,20,30) days, *κ* was selected by the formula given in (8) and the credible bounds were set at 5% and 97.5% giving an “in‐control” median run length of 210. For the STRAND Chart with a gate at 1/2 day, *κ* was again selected by the formula given in (8) and the credible bounds were set at 13.6% and 97.5%. For the uEWMA with LLR scores, all parameters were set at the values specified in the paper of Steiner and Jones,^14^ except the upper signalling boundary that was set at *h*_*u*_ = 0.009. For the uEWMA with scores relating to 30‐day survival, *κ* was set at 0.01, the lower reflecting boundary was set at *h*_*l*_ = 0.01, the upper boundary at *h*_*u*_ = 0.048, and the chart was initialised at *S*_0_ = 0.025.

It makes intuitive sense that the STRAND Chart is more comparable in terms of performance to the uEWMA with scores relating to 30‐day survival, it being based on n‐day survival rates, than to the uEWMA with LLR scores. It is possible to run the STRAND Chart as a set of EWMAs or CUSUMs with LLR scores and it is likely that, in that case, the performance would be more alike to the uEWMA with LLR scores, though this is a hypothesis we have not formally tested. We recommend using RA EWMAs for the strands, however, as this provides a direct way of estimating features of interest of the process. Where LLR scores are more optimal, scores relating to summary measures of the survival distribution, such as tail areas, are more interpretable. LLR scores also require a fully specified survival model for the data and specification of the alternative hypothesis to be tested for.

## DISCUSSION

The STRAND Chart for online monitoring of survival outcomes in continuous time keeps patient ordering intact, as the uEWMA,^14^ Weighted CUSUM,^21^ and Improved Bernoulli CUSUM^10^ do. The Biswas and Kalbfleisch continuous‐time Cox proportional hazards CUSUM does not keep patient ordering intact in the same way,^16^ as we have argued, but does update as and when information comes in and so is more sensitive to recent change in the process being monitored.

As in the papers of Steiner and Jones, Yashchin, and Keefe et al,^10^, ^14^, ^21^ patient ordering according to real‐time ordering of surgery is maintained, but here, short survival times do not affect all strands until the censoring period is up and all survival times for that cohort of patients who are operated on the same day (or on neighbouring days) have been collected. Thus, the short survival times do not get larger weights than the longer survival times in the same cohort simply because they are more contemporary. Also, the longer survival times are not lost in the chart, since the chart in effect waits for them to come through and to be weighted fairly against the short times. The layered nature of the chart and the natural real‐time delay mean that short‐term survival patterns affect the lower strands of the chart, where these relate to short‐term survival rates, earlier in real monitoring time than they affect the upper strands of the chart, where these relate to longer‐term survival rates (within the censoring window).

The STRAND chart can be applied within a Bayesian framework, so is akin to Bayesian changepoint methods for monitoring survival outcomes,^39^ but, in contrast to those methods, is an online as opposed to retrospective method. The methods we review in this paper are, like the STRAND chart we present, also online methods.^14^, ^15^, ^16^, ^21^ Where online methods can detect process change quickly, retrospective methods are ideally used as tools for locating the time of change (or changepoint).

We do not try to discuss in full the relative merits of the Classical and Bayesian approach to applying the EWMA here, as it is beyond the scope of this paper. For more insight, there is a background of available literature.^11^, ^17^, ^28^, ^29^, ^30^, ^31^, ^32^, ^33^, ^42^

The basic form of the STRAND Chart that we present is semiparametric in nature, much like the Cox proportional hazards CUSUM of Biswas and Kalbfleisch^16^ in that the survival distribution is not fully specified. However, any statistic could be used on each of the strands, in particular, the more efficient LLR CUSUMs or log hazard ratio CUSUMs. We present the chart as a collection of RA EWMAs as this provides semiparametric smoothed estimates of percentiles of the survival distribution that are optimal time series smoothers.^43^ Fully parametric methods have less freedom, and CUSUMs based on the likelihood, although optimal in terms of efficiency, do not immediately give smoothed estimates of features of the survival distribution.

In the example application to the now classic UK post cardiac surgery dataset of 1992‐1997, we show that the STRAND Chart can describe the nature of clusters of failures as they ripple through the chart, and we highlight where unusually long survivals of patients are balancing out the unusual failures. Previously, undetected features of the data are highlighted by the chart. Through bootstrapping of this dataset, we show that, given the format of estimating n‐day survival, the performance of the STRAND Chart is closer to that of the uEWMA with scores relating to 30‐day survival than to the more optimal uEWMA with LLR scores. We also show that STRAND Charts with a single early strand might be more efficient for detecting process change but we note that such a chart would perhaps not describe the survival process in enough detail. We expect that a STRAND Chart based on CUSUMs would have efficiency more alike to that of the uEWMA with LLR scores. This is potential area for future investigation.

## Supporting information

SIM8065‐Supp‐0001‐code_description.pdf

SIM8065‐Supp‐0002‐strand_0.1‐1.tar.gz

## References

[1] Bartroff J , Lai TL , Shih M . Sequential Experimentation in Clinical Trials: Design and Analysis. New York, NY: Springer Science & Business Media; 2012.

[2] Whitehead J . The Design and Analysis of Sequential Clinical Trials. Chichester, UK: John Wiley & Sons; 1997.

[3] Whitehead J , Jones D . The analysis of sequential clinical trials. Biometrika. 1979;66(3):443‐452.

[4] Spiegelhalter D , Sherlaw‐Johnson C , Bardsley M , Blunt I , Wood C , Grigg O . Statistical methods for healthcare regulation: rating, screening and surveillance. J R Statist Soc A. 2012;175(1):1‐47.

[5] Woodall WH , Grigg OA , Burkom HS . Research issues and ideas on health related surveillance In: Frontiers in Statistical Quality Control. Vol‐9 Berlin, Germany: Physica‐Verlag; 2010:145‐155.

[6] Woodall WH , Mohammed MA , Lucas JM , Watkins R , et al. The use of control charts in health‐care and public‐health surveillance. Discussion/Rejoinder. J Qual Technol. 2006;38(2):89‐134.

[7] Spiegelhalter D , Grigg O , Kinsman R , Treasure T . Risk‐adjusted sequential probability ratio tests: applications to Bristol, Shipman and adult cardiac surgery. Int J Qual Health Care. 2003;15(1):7‐13. 12630796 10.1093/intqhc/15.1.7

[8] Montgomery DC . Statistical Quality Control.New York, NY: Wiley New York; 2009.

[9] Shewhart WA . Economic Control of Quality of Manufactured Product. Milwaukee, WI: ASQ Quality Press; 1931.

[10] Keefe MJ , Loda JB , Elhabashy AE , Woodall WH . Improved implementation of the risk‐adjusted Bernoulli CUSUM chart to monitor surgical outcome quality. Int J Qual Health Care. 2017;29(3):343‐348. 28444331 10.1093/intqhc/mzx036

[11] Grigg OA . Risk‐Adjusted Monitoring and Smoothing in Medical Contexts [PhD thesis]. Cambridge, UK: University of Cambridge; 2004.

[12] Steiner SH , Cook RJ , Farewell VT , Treasure T . Monitoring surgical performance using risk‐adjusted cumulative sum charts. Biostatistics. 2000;1(4):441‐452. 12933566 10.1093/biostatistics/1.4.441

[13] Zhang X , Woodall WH . Dynamic probability control limits for risk‐adjusted Bernoulli CUSUM charts. Statist Med. 2015;34(25):3336‐3348. 10.1002/sim.6547 26037959

[14] Steiner SH , Jones M . Risk‐adjusted survival time monitoring with an updating exponentially weighted moving average (EWMA) control chart. Statist Med. 2010;29(4):444‐454. 10.1002/sim.3788 19908262

[15] Sego LH , Reynolds Jr MR , Woodall WH . Risk adjusted monitoring of survival times. Statist Med. 2009;28(9):1386‐1401. 10.1002/sim.3546 19247982

[16] Biswas P , Kalbfleisch JD . A risk‐adjusted CUSUM in continuous time based on the Cox model. Statist Med. 2008;27(17):3382‐3406. 10.1002/sim.3216 18288785

[17] Grigg OA , Spiegelhalter DJ . A simple risk‐adjusted exponentially weighted moving average. J Am Statist Ass. 2007;102(477):140‐152.

[18] Grigg OA , Farewell VT . An overview of risk‐adjusted charts. J R Statist Soc Series A. 2004;167(3):523‐539.

[19] Lovegrove J , Sherlaw‐Johnson C , Valencia O , Treasure T , Gallivan S . Monitoring the performance of cardiac surgeons. J Oper Res Soc. 1999;50(7):684‐689.

[20] Poloniecki J , Valencia O , Littlejohns P . Cumulative risk adjusted mortality chart for detecting changes in death rate: observational study of heart surgery. Br Med J. 1998;316:1697‐1700. 9614015 10.1136/bmj.316.7146.1697 PMC28566

[21] Yashchin E . Design and implementation of systems for monitoring lifetime data In: Frontiers in Statistical Quality Control. Vol 10 Berlin, Germany: Physica‐Verlag; 2012:171‐195.

[22] Yashchin E . Computational and Monte‐Carlo aspects of systems for monitoring reliability data In: Proceedings of COMPSTAT'2010. Berlin, Germany: Physica‐Verlag; 2010:253‐262.

[23] Page ES . Continuous inspection schemes. Biometrika. 1954;41:100‐115.

[24] Hunter JS . The exponentially weighted moving average. J Qual Technol. 1986;18(4):203‐210.

[25] Roberts SW . Control chart tests based on geometric moving averages. Technometrics. 1959;1(3):239‐250.

[26] Sonesson C . Evaluations of some exponentially weighted moving average methods. J Appl Stat. 2003;30(10):1115‐1133.

[27] Lucas JM , Saccucci MS . Exponentially weighted moving average control schemes: properties and enhancements. Technometrics. 1990;32(1):1‐12.

[28] Vidoni P . Exponential family state space models based on a conjugate latent process. J R Statist Soc B. 1999;61(1):213‐221.

[29] Grunwald GK , Hamza K , Hyndman RJ . Some properties and generalizations of non‐negative Bayesian time series models. J R Statist Soc Some B. 1997;59(3):615‐626.

[30] West M , Harrison PJ . Bayesian Forecasting and Dynamic Models. 2nd ed. New York, NY: Springer‐Verlag; 1997.

[31] Pole A , West M , Harrison PJ . Applied Bayesian Forecasting and Time Series Analysis. London, UK:Chapman & Hall/CRC; 1994.

[32] West M , Harrison PJ , Migon HS . Dynamic generalized linear models and Bayesian forecasting. J Am Statist Ass. 1985;80(389):73‐83.

[33] Smith JQ . A generalization of the Bayesian steady forecasting model. J R Statist Soc B. 1979;41(3):375‐387.

[34] Harrison PJ , Stevens CF . Bayesian forecasting (with discussion). J R Statist Soc B. 1976;38:205‐247.

[35] Colosimo BM . Bayesian control charts In: Encyclopedia of Statistics in Quality and Reliability. Hoboken, NJ: John Wiley & Sons, Inc; 2008.

[36] Cox DR . Regression models life tables. J R Statis Soc Series B. 1972;34(2):187‐220.

[37] Yashchin E . Weighted cumulative sum technique. Technometrics. 1989;31(3):321‐338.

[38] Assareh H , Mengersen KL . Estimation of the time of a linear trend in monitoring survival time. Health Serv Outcomes Res Methodol. 2014;14(1):15‐33.

[39] Assareh H , Mengersen KL . Change point estimation in monitoring survival time. PloS One. 2012;7(3):e33630. 22438969 10.1371/journal.pone.0033630 PMC3306432

[40] Assareh H , Mengersen KL . Bayesian estimation of the time of a decrease in risk‐adjusted survival time control charts. IAENG Int J Appl Math. 2011;41(4):360‐366.

[41] Parsonnet V , Dean D , Bernstein AD . A method of uniform stratification of risks for evaluating the results of surgery in acquired adult heart disease. Circulation. 1989;779(1):1‐12. 2720942

[42] Key PB , Godolphin EJ . On the Bayesian steady forecasting model. J R Statist Soc B. 1981;43(1):92‐96.

[43] Muth JF . Optimal properties of exponentially weighted forecasts. J Am Statist Ass. 1960;55(2):299‐306.

