# Supplementary material for: The STRAND Chart: A survival time control chart
Source: Stat Med. 2018 Dec 26;38(9):1651–61. doi: 10.1002/sim.8065 (PMC6767103; doi:10.1002/sim.8065)
Supplement: Supplementary file 1 — SIM8065‐Supp‐0001‐code_description.pdf [file SIM-38-1651-s001.pdf]

Package ‘strand’

November 15, 2018

Title Survival Time Risk-Adjusted N-Division Charts

Version 0.1.1

Description The STRAND Chart is an online monitoring tool for quality control and surveillance of survival or duration data. Binary gates track the progress of a patient or component through time. Each strand provides an estimate of failure probability at the relevant gate for a standard patient or component. There are N gates g1,g2,...,gN and the chart plotted consists of N Bernoulli RA EWMAs (Risk-adjusted Exponentially Weighted Moving Averages) with Beta credible intervals.

Depends R (>=3.2.0)

Imports stats, graphics

Suggests MASS

License GPL-3

LazyLoad yes

LazyData yes

NeedsCompilation yes

Author Olivia Grigg [cre, aut]

Maintainer Olivia Grigg <mathsworkmusic@gmail.com>

Repository Statistics in Medicine

Date/Publication 2018-11-15 16:00:00 UTC

R topics documented:

strand-package 1
strand.data.frame 2
strand.chart 2

Code 4

`strand-package` *Draws survival time risk-adjusted N-division charts*

Description

The strand package can be used to draw STRAND Charts, which are an online monitoring tool for quality control and surveillance of survival or duration data.

Details

The most important functions are `strand.data.frame` and `strand.chart`.

strand.data.frame   *Create STRAND object*

Description

Creates STRAND object from survival or duration data

Usage

```
strand.data.frame(data,gates,score = 0,baseline_score = 0,pilot_cutoff = 1,
                  times = 1:length(data),p0 = NULL,s = NULL,k = NULL,ph = NULL,
                  lower = 0.025,upper = 0.975,tol_u = 0.99999,tol_d = 0.00001)
```

Arguments

`data`	a vector of survival times
`gates`	a vector of cut-off times
`score`	a vector of risk scores to accompany survival times (default 0)
`baseline_score`	scalar baseline risk score
`pilot_cutoff`	number of data used as pilot data
`times`	calendar initialisation times to accompany survival times (default 1:length(data))
`p0`	initial probability of failure (default NULL)
`s`	risk coefficient (default NULL)
`k`	EWMA smoothing parameter (default NULL)
`ph`	target probability of failure (default NULL)
`lower`	lower percentage tail of credible interval (default 0.025)
`upper`	upper percentage tail of credible interval (default 0.975)
`tol_u`	upper tolerance of EWMA
`tol_d`	lower tolerance of EWMA

Value

an object of class ‘strand.data.frame’

strand.chart   *Draw a STRAND chart*

Description

Draw a STRAND chart

Usage

```
strand.chart(data,gates,lth=length(data),pilot_cutoff = 1,times = 1:length(data),
        pilot_time = times[pilot],strand_output,ph = strand_output[(3*lth+7),],
        yaxis_stretch = 2.9,lab_stretch_u = 1,lab_stretch_d = 0.9,cex = 1,
        cex.axis = 1,cex.lab = 1,cex.sig = 1,save = FALSE,width = 9,
        height = 12,family = "Palatino")
```

Arguments

`data`	a vector of survival times
`gates`	a vector of cut-off times
`pilot_cutoff`	number of data used as pilot data
`times`	calendar initialisation times to accompany survival times (no default)
`pilot_time`	calendar time of pilot data cut-off
`strand_output`	an object of class `strand.data.frame`
`ph`	target probability of failure (default `strand_output[(3*lth+7),]`)
`yaxis_stretch`	stretch factor of y-axis (default 2.9)
`lab_stretch_u`	stretch factor of upper signalling labels (default 1)
`lab_stretch_d`	stretch factor of lower signalling labels (default 0.9)
`cex`	size of plotting symbols (default 1)
`cex.axis`	size of axis text (default 1)
`cex.lab`	size of axis labels (default 1)
`cex.sig`	size of signalling labels (default 1)
`save`	logical indicating save to file
`width`	width of output graph
`height`	height of output graph
`family`	font family of text

Value

a graph of a STRAND Chart

R Code

Sub-functions

```
p_risk<-function(r,s,u_s){                       #Function to calculate risk
          exp(r+s*u_s)/(1+exp(r+s*u_s))    #given score ‘u_s’ and current baseline
                                           #log odds risk ‘r’ and coefficient ‘s’
}

failure_ind = function(x,g){               #failures within gates indicator
   d <-matrix(0,length(x),length(g))
   for (i in 1:length(g)){
     d[x<=g[i],i] <-1
   }
   d
}

ra_surv_times = function(x,g,u,b,ub){      #risk-adjusted survival times
 xb     <-x*exp(b*u)/exp(b*ub)
 xb_mat <-matrix(rep(xb,length(g)),length(g),length(xb)) #as a matrix
 xb_mat
}

logistic.pilot = function(x,g,u,ub,pilot){ #logistic regression output
  p0 <-0
  s  <-0
  u_s <-u - ub
  x   <-x[1:pilot]
  u_s <-u_s[1:pilot]
  for (i in 1:length(g)) {
    d <-failure_ind(x,g[i])
    logistic <-glm(d ~ u_s, family = binomial)
    s[i]  <-logistic$coef[2]
    p0[i] <-p_risk(logistic$coef[1],s[i],0)
  }
  output <-rbind(p0,s)
}
```

Function to create STRAND object

```
strand.data.frame = function(x,g,u,ub,pilot = 1,times = 1:length(x),p0 = NULL,s = NULL,k = NULL,
                             ph = NULL,lower = 0.025,upper = 0.975,tol_u = 0.99999,
                             tol_d = 0.00001){

 if (is.null(p0)) p0 <- logistic.pilot(x,g,u,ub,pilot)[1,]
```

```
if (is.null(s))  s  <- logistic.pilot(x,g,u,ub,pilot)[2,]
if (is.null(k))  k  <- 0.993 - 0.192*p0
if (is.null(ph)) ph <- p0

u_s <-u -ub

d <-matrix(0,length(x),length(g)) #failures within gates indicator
for (i in 1:length(g)) {
  d[x<=g[i],i] <-1
 }

r0 <-log(p0/(1-p0)) #Logit transformation of failure vector

mu  <-matrix(p_risk(r0,s,0),1,length(g)) #initialising ewma matrix at estimated rate for centred
                                          #baseline level (0)

lab_d  <-rep(0,length(g)) #initialising lower signal indicator
lab_u  <-rep(0,length(g)) #initialising upper signal indicator
labd   <-0
labu   <-0
labdj  <-0
labuj  <-0

bk  <-1/(1-k)       #precision parameter (steady state precision of EWMA under assumptions)

hup <-matrix(qbeta(upper,mu*bk,(1-mu)*bk),1,length(g))  #initialising 99% beta credible bounds
hdn <-matrix(qbeta(lower,mu*bk,(1-mu)*bk),1,length(g))  #initialising 1% beta credible bounds

lth <-length(x)     #full data length

for(i in 1:lth){ z   <-d[i,]   #current failure outcome
                 xi  <-x[i]    #survival time (used if fails before gate)
                 y   <-u_s[i]  #risk score for current outcome #lowest poss value must be zero
                 m        <-pmin(mu[i,],tol_u) #current value on mean scale
                 m        <-pmax(m,tol_d)
                 odds     <-m/(1-m)            #current value on odds scale
                 mu_new   <-k*m+(1-k)*(z-p_risk(log(odds),s,y)+m) #ewma with unconditional update
                 mu       <-rbind(mu,mu_new)          #chart vector value at time i
                 m        <-pmin(mu[i+1,],tol_u)      #next (forecasted) current value
                 m        <-pmax(m,tol_d)
                 hup      <-rbind(hup,qbeta(upper,m*bk,(1-m)*bk)) #upper credible bound vector
                 hdn      <-rbind(hdn,qbeta(lower,m*bk,(1-m)*bk)) #lower credible bound vector
                 for (j in 1:length(g)) {
                   if (i>pilot & (hdn[i+1,j]-ph[j])>0 & lab_u[j]==0) lab_u[j]<-times[i]+g[j]
                                                                   #labelling first u/l signal
                   if (i>pilot & (hup[i+1,j]-ph[j])<0 & lab_d[j]==0) lab_d[j]<-times[i]+g[j]
                 }
                 if (any(lab_u!=0)) {
                   labu  <-min(lab_u[lab_u>0])
                   labuj <-which(lab_u==min(lab_u[lab_u>0]))[1]
                 }
                 if (any(lab_d!=0)) {
                   labd  <-min(lab_d[lab_d>0])
                   labdj <-which(lab_d==min(lab_d[lab_d>0]))[1]
                 }
}
```

```
 if (length(g)>1) {
   output <-rbind(mu[2:(lth+1),],hup[2:(lth+1),],hdn[2:(lth+1),],lab_u,lab_d,rep(labu,dim(mu)[2]),
                rep(labd,dim(mu)[2]),rep(labuj,dim(mu)[2]),rep(labdj,dim(mu)[2]),ph)
 }
 if (length(g)==1) {
   output <-c(mu[2:(lth+1),],hup[2:(lth+1),],hdn[2:(lth+1),],lab_u,lab_d,labu,labd,labuj,labdj,ph)

 }
 output
}
```

Function to draw STRAND Chart

```
 strand.chart = function(x,g,lth=length(x),pilot = 1,times,pilot_time = times[pilot],
                         strand_output,ph = strand_output[(3*lth+7),],
                         yaxis_stretch = 2.9,lab_stretch_u = 1,lab_stretch_d = 0.9,cex = 1,
                         cex.axis = 1,cex.lab = 1,cex.sig = 1,save = FALSE,width = 9,
                         height = 12,family = "Palatino"){

 mu  <-strand_output[1:lth,]
 hup <-strand_output[(lth+1):(2*lth),]
 hdn <-strand_output[(2*lth+1):(3*lth),]
 lab_u <-strand_output[(3*lth+1),]
 lab_d <-strand_output[(3*lth+2),]
 labu  <-strand_output[(3*lth+3),1]
 labd  <-strand_output[(3*lth+4),1]
 labuj <-strand_output[(3*lth+5),1]
 labdj <-strand_output[(3*lth+6),1]


  if (save==FALSE | save==F) X11() #opening in R graphics window
  if (save==TRUE | save==T) postscript("strand_chart.ps",paper="special",width=width,
                                       height=height,horizontal=FALSE,onefile=FALSE,
                                       family=family) #writing to postscript file
  par(mfrow=c(length(g),1))
  par(mar=c(2.5,4,0,4)+0.1)
  par(mgp=c(1.4,0.7,0))
  for(j in length(g):1){
    plot(times+g[j],mu[,j],pch="",type="l",xlab="time",ylab="p",
         ylim=c(max(0,-0.2*median(hup[,j])),min(1,yaxis_stretch*median(hup[,j]))),
         cex=cex,cex.lab=cex.lab,cex.axis=cex.axis,lwd=0.8)
    polygon(c(times+g[j],rev(times)+g[j]),c(hdn[,j],rev(hup[,j])),density=100,
          col="darkgrey")                     #shaded from upper to lower credible bounds
    abline(v=pilot_time,lty=2)                #pilot data cut-off
    lines(times+g[j],mu[,j],lwd=0.8)
    if(lab_u[j]!=0) text(0.1*pilot_time,lab_stretch_u*yaxis_stretch*median(hup[,j]),
                         lab_u[j],col="indianred3",cex=cex.sig,pos=1)
      #marking upper signals at each gate
```

```
    if(lab_d[j]!=0) text(0.1*pilot_time,lab_stretch_d*yaxis_stretch*median(hup[,j]),
                         lab_d[j],col="turquoise4",cex=cex.sig,pos=1)
      #marking lower signals at each gate
    text(max(times),lab_stretch_u*yaxis_stretch*median(hup[,j]),paste("G:",j),cex=1.2,
         pos=1,col="darkgrey")
    points(times[which(hdn[,j]>ph[j])]+g[j],hup[,j][which(hdn[,j]>ph[j])],pch=16,
           col="indianred3",cex=0.45)
    points(times[which(hup[,j]<ph[j])]+g[j],hdn[,j][which(hup[,j]<ph[j])],pch=16,
           col="turquoise4",cex=0.3)
   }
    if (save==TRUE | save==T) dev.off()

}
```
